# Electric Potential Profiles in a Model Single-Path Electrodialysis Unit

**DOI:** 10.3390/membranes12111136

**Published:** 2022-11-12

**Authors:** Jan Pagáč, Petr Kovář, Zdeněk Slouka

**Affiliations:** 1 Department of Chemical Engineering, University of Chemistry and Technology Prague, Technická 3, 16628 Prague, Czech Republic; 2 New Technologies-Research Centre, University of West Bohemia, Univerzitní 8, 30614 Pilsen, Czech Republic

**Keywords:** electrodialysis, electric potential, diluate, overlimiting current, desalination

## Abstract

Electrodialysis is an important electromembrane separation process anticipated to play a significant role in developing future technologies. It produces ion-depleted and ion-concentrated product streams, intrinsically suggesting the formation of spatial gradients of relevant quantities. These quantities affect local conditions in an electrodialysis unit. To investigate the spatial distribution of electric potentials, we constructed a model electrodialysis system with a single diluate channel that included ports for inserting reference electrodes measuring potential profiles. We validated our system and measurement methods in a series of control experiments under a solution flow rate of 250 µL/min and current densities between 10 and 52 A/m^2^. The collected data showed that the electric potential in the diluate channel did not change in the vertical direction (direction of gravity force), and only minimally varied in the diluate channel center in the flow direction. Although we could not reconstruct the potential profile within ion-depleted layers due to the resolution of the method, we found appreciable potential variation across the diluate channel. The most significant potential drops were localized on the membranes with the developed ion-depleted zones. Interestingly, these potential drops abruptly increased when we applied current loads, yielding almost complete desalination. The increase in the resistance accompanied by relatively large fluctuations in the measured potential indicated the system transition into limiting and overlimiting regions, and the onset of overlimiting convection.

## 1. Introduction

Electrodialysis is classified as an electromembrane separation process. Other essential electromembrane techniques include electrodeionization, capacitive deionization, electrodialysis with bipolar membranes, or electrodialysis reversal [1]. Electromembrane processes separate ionic components from electrolyte solutions by applying an electric potential gradient. The principle, thus, relies on the electric charge borne by the individual components and the coupling of its transfer with mass transport. In general, the selectivity of separation is provided by permselective membranes.

Ion-selective membranes are vital to these processes, since they allow us to separate anions or cations. Ion-exchange membranes are categorized according to their selectivity as cation-exchange, anion-exchange, or bipolar. Their macroscopically observable structure divides them into homogeneous or heterogeneous [2]. Cation-exchange membranes contain negatively charged groups (e.g., –SO_3_^−^, –COO^−^, –PO_3_^2−^, –PHO_2_^−^, –AsO_3_^2−^, –SeO^−^) covalently fixed to the polymer matrix. Under no external electric field, these negative fixed charges are in local electric equilibrium with mobile cations, denoted as counterions. In this case, mobile anions carrying the same charge as the fixed groups in the membrane are called co-ions. Due to the repulsive electrostatic forces between membrane fixed charge and mobile co-ions, the cation-exchange membrane is impermeable to anions. Anion-exchange membranes containing a positive fixed charge (e.g., –N^+^H_2_R; –N^+^HR_3_; –N^+^R_3_; –P^+^R_3_; –S^+^R_2_) are preferentially permeable to anions [3,4,5]. The extent to which an ion-selective membrane is impermeable to co-ions is quantified by its permselectivity [6,7]. 

The permselectivity of ion-exchange membranes reflects in their nonlinear current–voltage curves showing three specific regions: ohmic (underlimiting), limiting, and overlimiting [8]. The first (underlimiting) region is characterized by a linear current growth with voltage, rendering the system a resistor. Here, diffusion and migration are the predominant ion transport mechanisms across the membrane. In the vicinity of the membrane, however, the selective ionic transport [9] produces ion-poor and ion-rich zones. This phenomenon is regarded as concentration polarization and leads to the development of ion-depleted and ion-concentrated zones [10]. The depletion and concentration of ions stipulate local electrolyte conductivity, which, in turn, dictates the strength of the local electric field and, thus, electric potential distribution. Using a unique milifluidic system, we evaluated the local electric field to be tens of kV/m and hundreds of V/m in the ion-depleted and ion-concentrated zones [11]. The ion-depleted zones increase the system resistance and, later, dictate the behavior of the ion-exchange membrane. In an electromembrane stack, the ion-depleted and ion-concentrated membrane sides correspond to the dilute and concentrate sides, respectively. The concentration polarization, thus, increases the energy consumption to achieve the same desalination degree. At a specific polarization voltage (or polarization current), the ionic concentration at the ion-depleted zone and membrane interface reaches virtually zero, a moment translating the system into the limiting region. The current at which this transition occurs is called the limiting current [12]. With further voltage increases, the passing current tends to level off. The actual current saturation independent of the polarization voltage is predicted by a theory based on the Nernst–Planck equation, assuming local electroneutrality and neglecting convective contribution [13]. According to this theory, further voltage increase extends the ion-depleted regions, accompanied by resistance growth.

In most cases, however, the limiting value of the electric current is exceeded, and the current–voltage curve displays the third, overlimiting region. The unexpected overlimiting ionic transport was a mystery when revealed. In the last few decades, experimental and numerical work has attributed this phenomenon to a myriad of reasons, such as electroconvection, water splitting, natural convection, or changes in ion selectivity [14,15,16,17]. Electroconvection forms stable vortices, mixing the ion-depleted region with the treated solution bulk [18]. The existence of these vortices has been confirmed by theoretical analysis [19,20], numerical solution of the Navier–Stokes and Poisson–Nernst–Planck equations [21,22], and experimentally [8,23,24,25]. The origin of vortices is in electric forces developing in the ion-depleted zone due to the interaction of a strong local electric field and space charge regions [26,27]. The geometry of ion-exchange membranes and their internal structure significantly affects the vortex appearance. Recent experimental work also revealed a strong effect of natural convection on the overlimiting regime [23,28]. At the same time, water splitting was observed at the anion-exchange membranes [29], probably owing to the catalytic activity of groups bound in the membrane [30].

In electrodialysis stacks, the diluate develops two ion-depleted zones at either membrane flanking the given channels. The operation of a membrane stack tackles the unwanted polarization effects by redirecting the pressure-driven flow; this is achieved by turbulizing meshes and employing relatively low current densities. The uncertainties associated with overlimiting polarization effects still prevent the electromembrane processes from operating in the high-current regimes [27], despite providing higher desalination degrees. Unfortunately, research in this area is hindered by the physical embodiment of the electrodialysis units that virtually allow one to treat the unit as a black box, i.e., the system behavior is inferred from the changes between the inlet and outlet. To our knowledge, no experimental work analyzing the internal (differential) variations of respective quantities along desalination channels, such as ionic concentrations, electric potentials, temperatures, etc., is publicly available. However, the formation and development of the diffusion layers and concentration profiles at ion-exchange membranes were studied using laser interferometry [31,32]. These profiles revealed the formation of ion-depleted and ion-concentrated zones at the ion-exchange membranes. The lack of experimental data or the impossibility of running experiments can be overcome by mathematical modeling. Although exciting, modeling ion transport through ion-exchange membranes still presents a rather tricky problem. The disparate length scales of electrodialysis units and electrostatic interactions in water solutions prevent the formulation of mathematical models on fundamental equations and require the incorporation of many approximations and simplifications. Numerical models have been used to investigate the influence of concentration polarization on ionic transport in simple membrane-electrolyte systems [33], or in electrodialysis and reverse electrodialysis [34,35]. Depending on the complexity of the models, they predict, e.g., the constant ionic bulk concentration of the processed solution and its decrease and increase in the diffusion boundary layer on the diluate and concentrate side, respectively [33,34,35]. The potential profiles are linear in the diffusion boundary layers and show a step potential variation in the membrane due to the fixed charge [33]. The linearity of potential profiles and the existence of constant bulk concentrations are examples of used simplifications.

To contribute to understanding membrane stack behavior for various degrees of desalination, we constructed a single-channel electrodialysis unit equipped with ports to measure electric potential profiles under desalination conditions. We measured electric potential profiles along and across the dilute channel and in the vertical direction. The measurements reveal the locations of the highest potential drops and, thus, the locations of the highest resistance for single-path desalination arrangement.

## 2. Materials and Methods

### 2.1. System Fabrication

**Figure 1:**
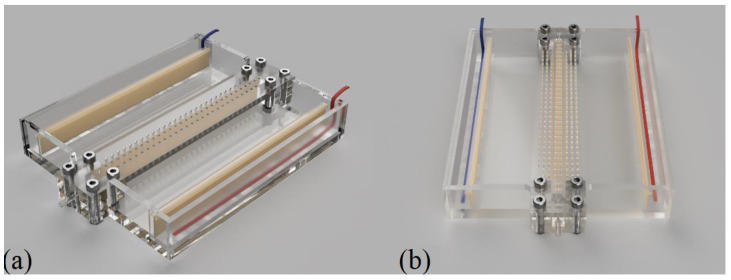
Three-dimensional schematics of completed electrodialysis systems (Unit 1—(**a**), Unit 2—(**b**)).

**Figure 2:**
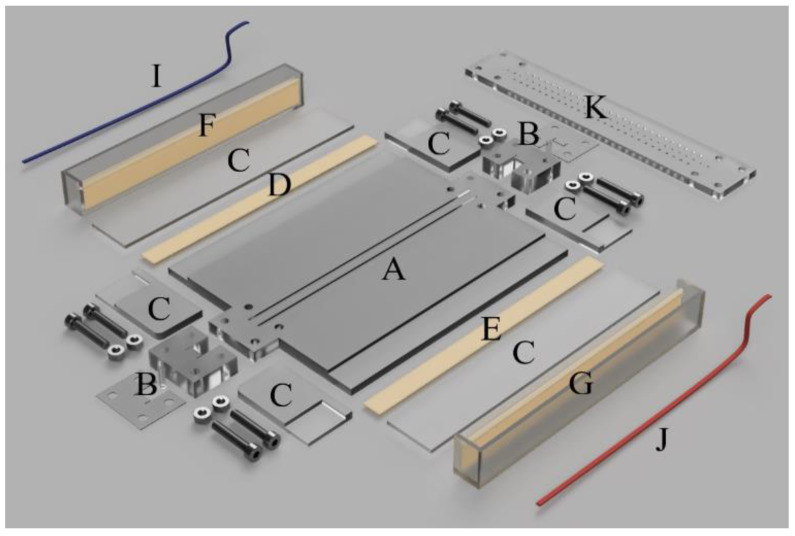
Illustration of a disassembled electrodialysis system; A—device base, B—input/output block with Tygon tubing for input/output and silicone seal, C—device walls, D—anion-exchange membrane, E—cation-exchange membrane, F—electrode compartment with anion-exchange membrane, G—electrode compartment with cation-exchange membrane, I—anode, J—cathode, K—the top wall of the device with measuring ports.

**Figure 3:**
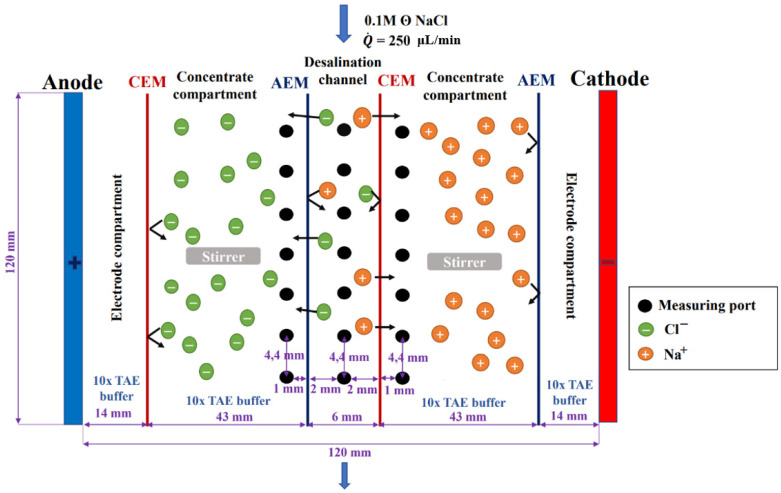
Schematic representation of Unit 1. Anions (green) and cations (orange) indicate the salt ions originating from the desalination channel.

We constructed two electrodialysis milifluidic systems, Unit 1 and Unit 2 (Figure 1a,b and Figure 2). The units were provided with rows of measuring ports on the depletion and concentration sides of the membranes. The main difference between Unit 1 and 2 was in the number of measuring ports (see below). Both units consisted of (i) a desalination channel sandwiched by heterogeneous cation- and anion-exchange membranes, (ii) two continuously stirred concentration compartments, and (iii) two electrode compartments (see Figure 3). The electrode compartments were separated from the concentrate compartment by another pair of membranes preventing bubbles or the ionic products of electrode reactions from affecting the measurements of potential profiles.

Both devices were made of polymethyl methacrylate (PMMA) plates purchased from Zenit a.s. (Czech Republic). The heterogeneous membranes sold under the brand name RALEX AM(H)-PES and CM(H)-PES were provided by MemBrain, a.s. (Stráž pod Ralskem, Czech Republic). The possibility of system disassembly allowed us to change the membranes in the system. 

All parts of an experimental unit are depicted in Figure 2. The overall dimensions of the system base (A) were 168 × 120 × 6 mm. Two 148 mm long grooves for inserting the membranes spaced 6 mm apart were milled into the center of the system base. The dimensions of the dilute channel were 140 mm in length, 6 mm in width, and 8 mm in height. The active membrane area was 120 × 8 mm^2^. The inlet and outlet walls consisted of two parts (B, C). The inlet part (B) had 4 holes to attach the upper wall with measuring ports (K) and two 4 mm deep grooves to insert the membranes. The sizes of the two concentrate and electrode (F, G) compartments were 140 × 43 mm^2^ and 140 × 14 mm^2^, respectively. The removable upper wall (K), of 168 × 30 × 4 mm^3^, also contained grooves for membrane insertion and sealing. In the case of Unit 1, 31 measuring ports (with a diameter of 1.5 mm) spaced 4.4 mm apart were located along the diluate channel, with 27 on each of the concentrate sides (see Figure 1a). These were placed 2 mm away from the membrane. 

In the case of the upper part of Unit 2, we made 3 rows of measuring ports between the membranes (in the desalination channel), totaling 93 ports (see Figure 1b). There were also 3 rows of measuring ports on the membrane concentrate sides. All PMMA parts were glued together using UV curable glue ACRIFIX^®^ 192. We glued two short pieces of Tygon tubing to the inlet and outlet holes, serving as inlet and outlet ports. These tubings had inner and outer diameters of 1.45 mm and 2.80 mm, respectively. The upper wall was mounted to the whole system with screws with silicone sealing placed at respective locations. 

### 2.2. Experimental Setup

The electrode and concentrate compartments were filled with 10x TAE buffer (Tris-Acetate-EDTA) to provide constant pH conditions in those compartments. An amount of 0.1 M of NaCl solution was processed in the desalination channel. The flow was applied by an AL-4000 piston pump (New Era Pump Systems Inc., Farmingdale, New York, NY, USA), which was connected to the ED system with Teflon tubing. The flow rate was set at 250 µL/min for all experiments, yielding a mean linear velocity of 0.52 cm/min. The concentrate compartments were stirred using Kamush (Zblewo, Poland) LPSm01-mini magnetic stirrers. The speed of the magnetic stirrers was set to 300 rpm. Platinum wires were inserted into the electrode compartments and connected to a 2400 SourceMeter^®^ (Keithley, Cleveland, OH, USA). This unit applied a fixed electric current for each measurement. The electrical potential values were measured using FLEXREF AgCl reference electrodes from WPI (Sarasota, Florida, USA) and were connected to 2601B SourceMeter^®^ (Keithley, Cleveland, OH, USA). Both units were connected to a desktop computer using a USB RS232 cable, which allowed continuous recording of the source voltage and measured electrical potential in the ED systems. The conductivity of the input and output solution was measured using a Horiba LAQUA Twin EC conductivity meter.

### 2.3. Measurement of Potential Profiles on Unit 1

Individual measurements of potential profiles were performed under fixed values of electric current. These values were 10 mA, 20 mA, 30 mA, 40 mA, and 50 mA, giving current densities of 10.42, 20.83, 31.25, 41.67, and 52.08 A/m^2^, respectively. Prior to the start of the measurements, the conductivity of the treated 0.1 M sodium chloride solution was measured. Subsequently, the piston pump flow rate was set to 250 µL/min, and a given current was applied. The device was left for approximately 25 min to reach a pseudo-steady state, indicated by a constant electric voltage at the power supply. The conductivity of the output solution was measured and recorded. Then, the actual measurement of the potential profiles began. A schematic representation of the measurement system is shown in Figure 3. The measurements were always taken at three heights of 0 mm (bottom of the dilute channel), 4 mm (center of the dilute channel), and 8 mm (just below the top wall of the diluate channel). The reference electrodes were fitted with non-conductive stops to set their proper measuring height. 

**Figure 4:**
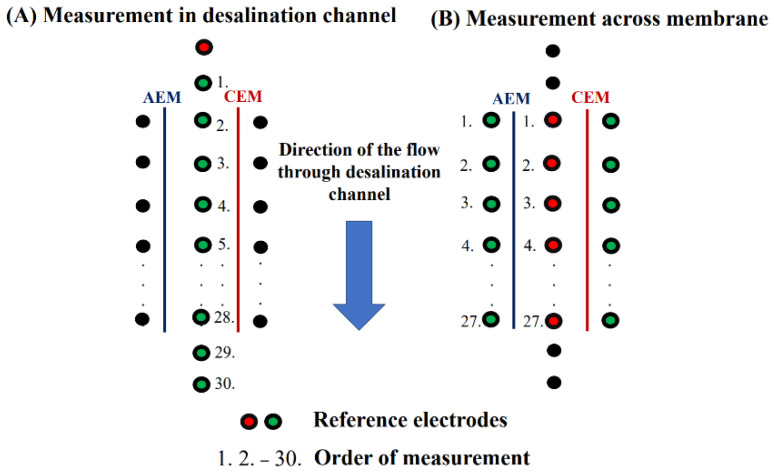
Schematics of the potential profile measurements: (**A**) along the desalination channel; (**B**) across the membranes.

To measure the potential profiles along the diluate channel, we placed one of the reference electrodes into the first measurement port of the diluate (red circle in Figure 4A) and the other successively into ports in the direction of the flow (green circles in Figure 4A). To test the robustness of our method, we changed the position of the first reference electrode and measured the profiles again. In this experiment, we inserted the first reference electrode into port number 15 and measured the potential profiles with respect to this position. The measurement of the potential profiles across the membranes proceeded according to the schematic in Figure 4B. The first electrode was sequentially placed into the middle ports in the flow direction (red circles in Figure 4B), and the other electrode into the adjacent ports in the concentrate compartments (green circles in Figure 4B). We obtained 27 electrical potential values for each membrane.

### 2.4. Measurement Procedure of Potential Profiles on Unit 2

**Figure 5:**
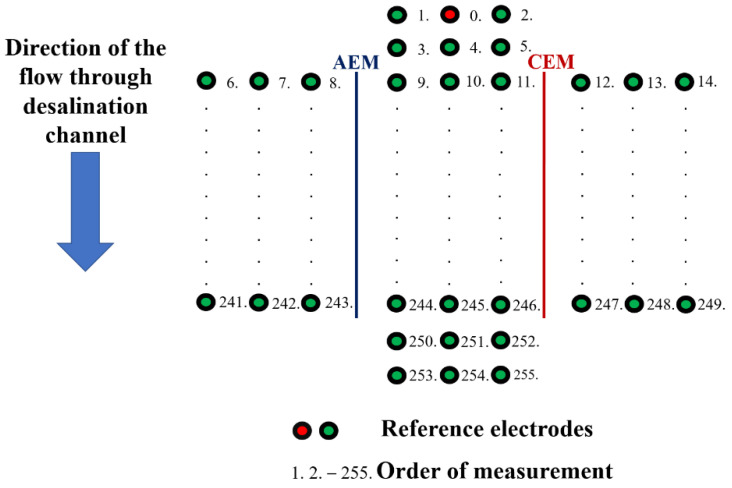
Schematics of the potential profile measurements on Unit 2.

The potential profiles measurement on Unit 2 was conducted with identical instruments and under identical flow rates, agitator speeds, and desalination channel geometries. The main difference is the number of measurement ports in the desalination channel and the side chambers. In Unit 2, we integrated 93 measuring ports directly along the diluate and 81 in each concentration compartment. The total number of measuring ports was 255. The experiments were carried out at three channel heights (at the bottom, middle, and top, as in Unit 1). The measurement started after reaching the pseudo-steady state. We selected the measurement electrode height, inserted the first reference electrode into the middle port in the first row (red circle in Figure 5), and measured potentials in the order given by the port numbers in Figure 5 by using the second electrode. This procedure was repeated for all three heights.

## 3. Results

### 3.1. Verification of Device Functionality 

Before measuring the potential profiles, verifying the desalination capabilities of the manufactured devices was necessary. For this reason, we conducted a parametric study that evaluated the overall desalination degree as a function of the applied current. This study was performed under the same operating conditions as the actual measurement of the potential profiles, i.e., under a constant flow rate of 250 µL/min of processed 0.1 M sodium chloride solution and with applied currents ranging from 0 to 50 mA in 5 mA steps. 

**Figure 6:**
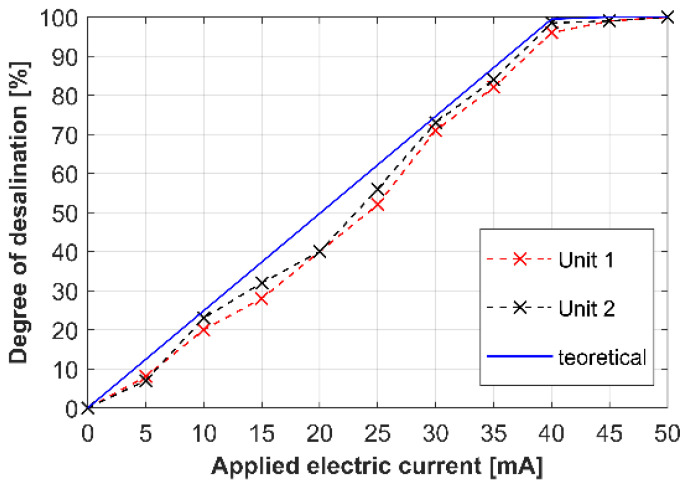
Desalination degree as a function of the applied current: Unit 1 (red), Unit 2 (black), theoretical desalination degree (blue).

The desalination degree was calculated using Equation (1), where c_in_ and c_out_ are the inlet and outlet solution concentrations. The concentrations were determined from the measured solution conductivities with experimental calibrations recalculating conductivities into concentrations. The resulting dependence of the degree of desalination on the applied current is shown in Figure 6. The red curve was obtained for Unit 1, and the black curve for Unit 2. The blue curve represents a theoretical prediction obtained from Equation (2). The theoretical concentration was evaluated from Equation (3), where c_out_, _theor_, c_in_, I, F, and Q_in_ are the theoretical outlet concentration, inlet concentration, electric current, Faraday constant, and volumetric flow rate. This equation assumes 100% ion selectivity of the membranes and 100% current efficiency in ion removal.

φ_s_ = (c_in_ − c_out_)/c_in_ · 100% (1)

φ_s,theor_ = (c_in_ − c_out,theor_)/c_in_ · 100% (2)

c_out,theor_ = c_in_ − I/(F · Q_in_) (3)

The results in Figure 6 show that the manufactured devices desalinated the processed solution according to the expectations. The desalination degree increased almost linearly with the increasing current up to 40 mA, at which point the output solution was almost completely desalted. The possibility to apply higher currents than that corresponding to the theoretical 100% desalination without a significant increase in the applied voltage indicated that the permselectivity of the membranes was lower than 100%. In other words, the current was carried by co-ions leaking through membranes with imperfect permselectivity. Since the complete solution desalination intrinsically imparts the conditions that are apt for developing overlimiting phenomena, we also focused on a possible contribution of water splitting to the overlimiting current. Water splitting generates new H^+^ and OH^−^ ions [36,37], increasing the conductivity in the system and attracting other ions into the ion-depleted zones through the exaltation effect [38]. In our previous work, we proved that water splitting proceeded on heterogeneous AEMs but not on heterogeneous CEMs [8,37]. Such asymmetric water splitting on membranes would lead to acidifying the diluate. However, the pH measurements in the diluate recovered from our system showed only insignificant changes in this quantity. Other overlimiting phenomena, such as electroconvection or natural convection, can contribute to the ion transport from the electrolyte core to the ion-depleted layer. At 100% desalination, however, the electrolyte bulk shall not contain those ions. As seen in Figure 6, we achieve a good agreement by comparing the experimental values of desalination (black and red curves) with the theoretically predicted ones (blue curve). The experimental curves lie slightly below the theoretical curve, also strongly pointing to the fact that the permselectivity of the membranes is indeed not 100%. The manufacturer of the used membranes, MemBrain a.s., reports permselectivity values of more than 90% [39]. The analysis of the desalination performance confirms the proper function of our desalination units.

### 3.2. Verification of the Measuring Method

**Figure 7:**
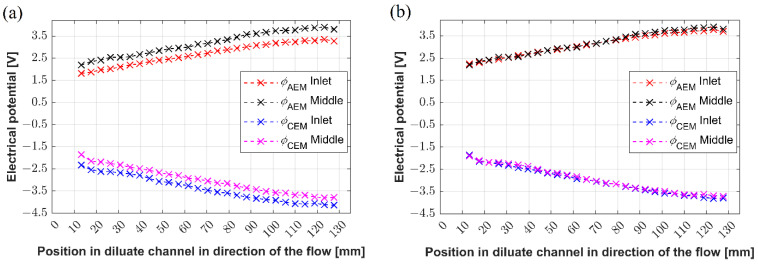
(**a**) The potential drops across the anion- and cation-exchange membranes established along the channel, (**b**) recalculated potential drops across the anion- and cation-exchange membranes established along the channel.

We tested the measuring method concerning its insensitivity to the position of the first reference electrode (the potentials are independent of the placement of the reference point). The first electrode is the electrode to which all measured potentials are referenced. We performed two experiments on Unit 1, in which the first electrode was placed either into the first or the middle port of the diluate channel. Then, we measured the potential drops across the cation-exchange and anion-exchange membranes. The results are plotted in Figure 7a. The magenta and black lines represent the potential values at CEM and AEM, respectively, when the first electrode was placed in the first port. The blue and red lines represent the potential values at CEM and AEM, respectively, when the first electrode was placed in the middle port. Both measured data sets show the same trend; however, they shifted approximately by 0.4 V. To clearly show that the corresponding curves are shifted by the potential difference of the two tested reference points, we replotted the data by adding the potential difference measured between the two reference points. The potential difference was equal to 0.43 V. As plotted in Figure 7b, the recalculated data nicely collapsed onto each other. 

The results described in this section document that our measurement method is robust and produces the same results irrespective of the reference electrode position.

### 3.3. Potential Profiles: Unit 1

**Figure 8:**
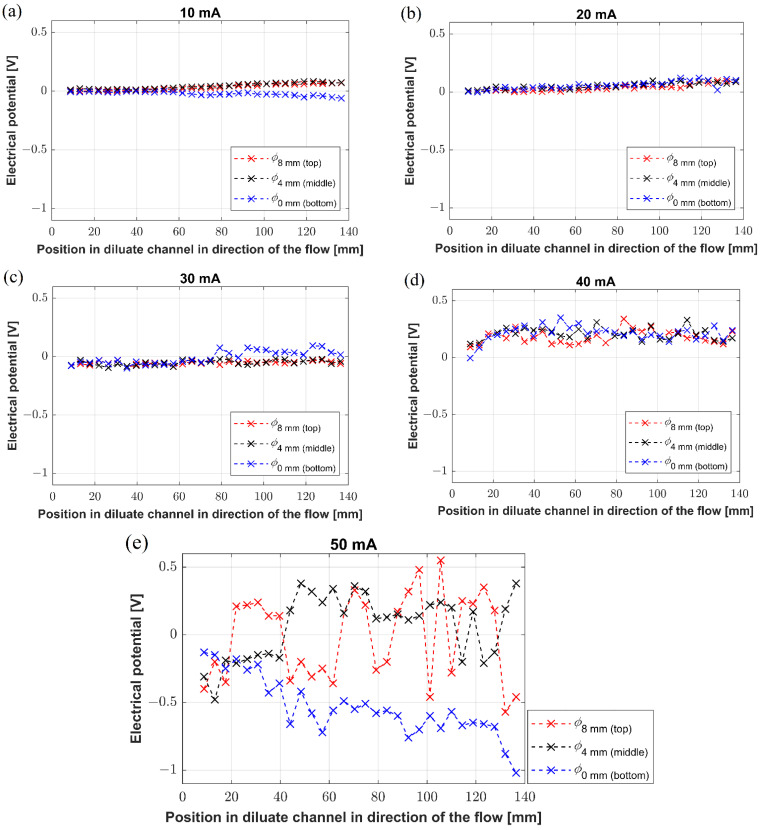
(**a**–**e**) The potential profiles established along the channel under various current loads.

The experiments with Unit 1 can be divided into two groups. The first group focused on reconstructing the potential profiles along the desalination channel at three different vertical levels, specifically at 0 mm, 4 mm, and 8 mm, measured from the bottom of the channel. The motivation for this measurement comes from our yet-unpublished data revealing that concentration changes occur along the diluate channel, as well as in the vertical direction (direction of gravity). Recently, we observed and PIV-tracked natural convection [25] in the membrane systems, suggesting the possible mechanism for generating concentration gradients in the vertical direction. Thus, the potential profiles measured in Unit 1 were measured to answer whether the concentration gradients affect the electric potential distribution as a function of the applied current load. The obtained potential profiles in the desalination channel are shown in Figure 8a–e. 

**Figure 9:**
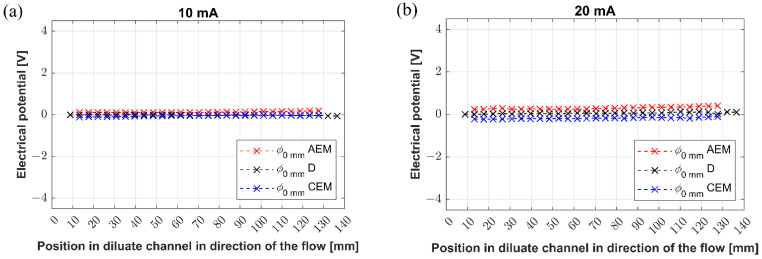

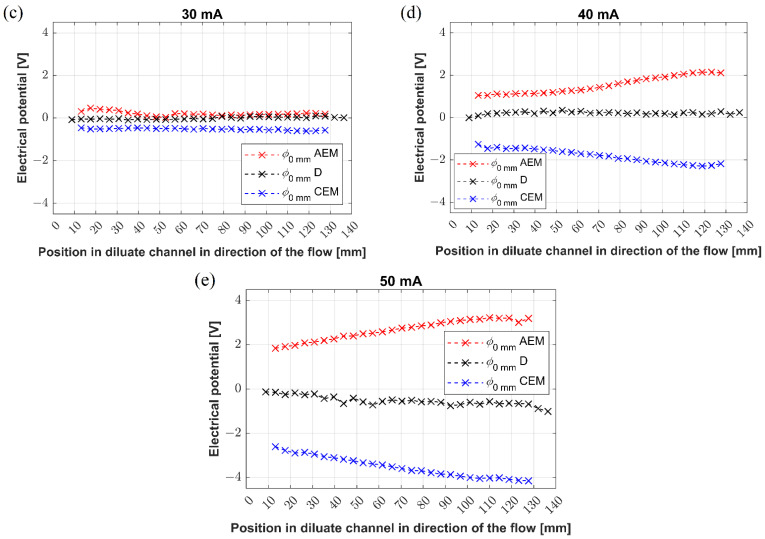
(**a**–**e**) The potential drops across membranes established along the channel under various current loads: AEM (red) potential drop across anion-exchange membrane, D (black) potential profile measured in diluate channel, CEM (blue) potential drop across cation-exchange membrane.

The second group of experiments investigated the potential drops across the AEM and CEM. This measurement was performed to reveal how the proceeding desalination resulting in concentration (and thus, conductivity) variations along the channel affects the potential distribution, and mainly, the potential drops across the particular membranes and the adjacent ion-depleted layers. The electrodes measured the profiles at the bottom of the system, i.e., at the channel height of 0 mm. The individual profiles from these experiments are shown in Figure 9a–e.

The electric potential profiles measured along the center of the desalination channel showed two characteristic qualities. First, the potential profiles did not vary with their vertical position, and second, they attained almost constant (although fluctuating at 50 mA) values along the whole channel (see curves in Figure 8a–e). The applied currents of 10 mA, 20 mA, and 30 mA (Figure 8a–c) produced virtually identical potential distributions, whose values were around zero. A displacement of the measuring electrode toward one of the membranes resulted in a change in the measured potential, which explains the off-zero trends and measured values. No potential changes in direction along the channel or with the vertical position are consistent with the fact that under those current loads, the system operates in an underlimiting regime with minimal possible interference of overlimiting mechanisms. The electric currents of 40 mA and 50 mA (Figure 8d,e) resulted in the potential profiles that were again independent of the position in the channel, i.e., the potential values did not change either along the channel or along the vertical axis. However, we observed large potential fluctuations given by large temporal variations at the given positions. The amplitude of fluctuations was proportional to the passing current, i.e., relatively small for 40 mA (tens of mV) and large for 50 mA (hundreds of mV). Both 40 and 50 mA produce solutions that are characterized by high desalination degrees appropriate for inducing overlimiting mechanisms, such as electroconvection and natural convection. As mentioned above, our previous work with heterogeneous membranes revealed strong convective streaming at both AEM and CEM under overlimiting conditions. This streaming is given by the simultaneous action of electroconvection and natural convection. The potential profiles may reflect fast vortices developing in the desalination channel, especially at 50 mA. 

Figure 9a–e shows the electric potential drops across the CEM and AEM and potential profile in the diluate channel measured at the bottom for the applied currents of 10 mA through 50 mA. Under low applied currents (10, 20, and 30 mA), the potential drops across the membranes are tens or hundreds of millivolts, and they slowly grow with the increasing current load. These drops are virtually constant along the channel. This observation again points to the underlimiting regime in the desalination channel. The situation completely changes for 40 and 50 mA when significant potential drops develop across both membranes. The potential profiles reflect the applied current load and correlate with the degree of desalination. The potential drops reach values of 1 and 2 V at the channel inlet for 40 and 50 mA, respectively, and monotonously grow to about 2 and 3 V at the channel output. The voltage losses across AEM are lower than those across CEM when referenced to the potential in the center of the diluate channel. One of the contributing factors to this behavior is the higher conductivity of AEM than CEM. The sudden growth of the potential drops across the membranes indicates that the conditions yielding complete desalination incur large resistances in the diluate. At the same time, large variations of the voltage drops across the membranes for larger currents imply that the current density also varies significantly along the channel. This is largest at the desalination channel inlet and smallest at the outlet. The variation in current density may also result in temperature gradients associated with Joule heat dissipation, which, in turn, may intensify natural convection at those locations. 

### 3.4. Potential Profiles: Unit 2

**Figure 10:**
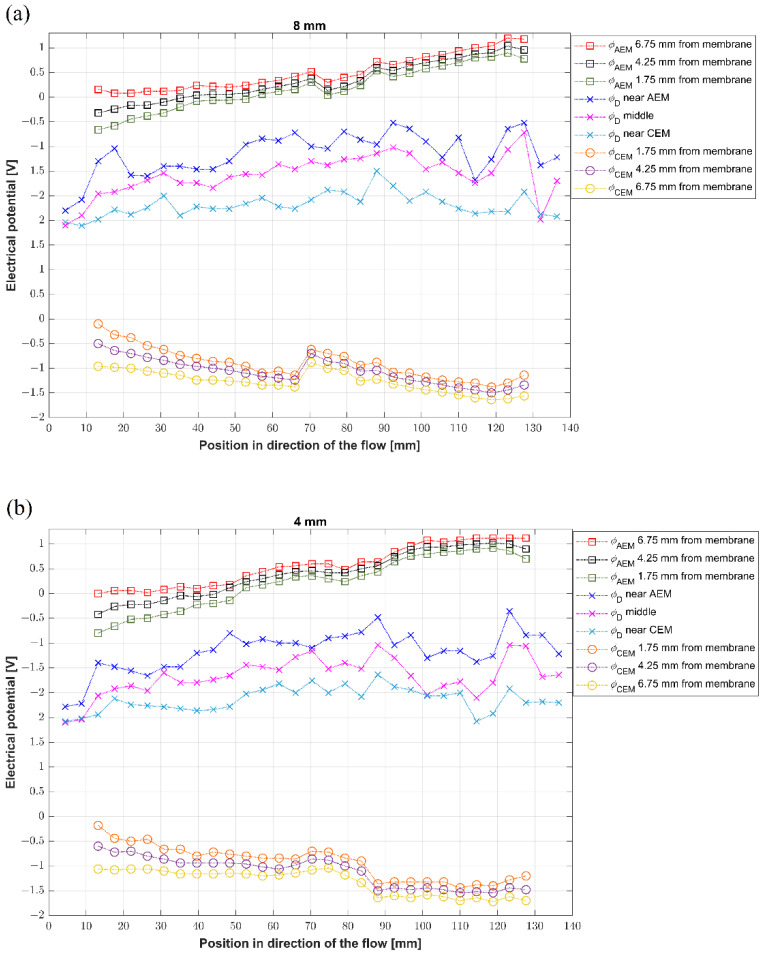

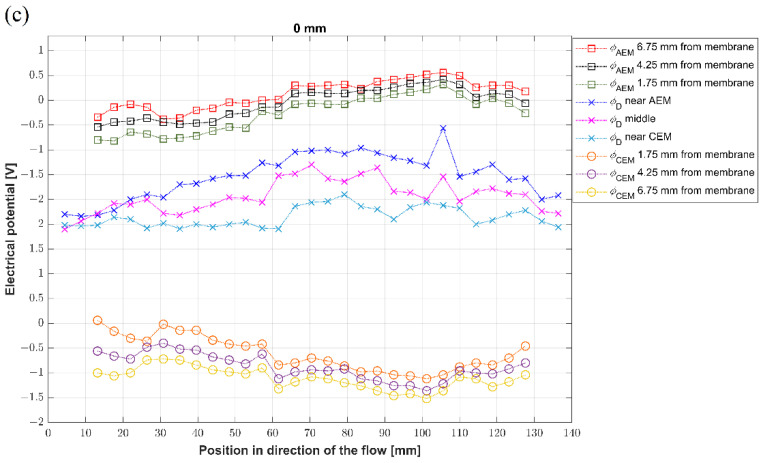
(**a**–**c**) The potential profiles established in horizontal planes along the channel: (**a**) top wall—8 mm, (**b**) middle—4 mm, (**c**) bottom—0 mm.

Unit 2 primarily served for a detailed reconstruction of the potential profiles in the desalination channel and on the concentration side of the membranes. Due to the high number of measuring ports, we performed this experiment at the current load of 40 mA, providing conditions that were sufficient for complete, single-path desalination. The results are shown in Figure 10a–c, in which pane (a) captures the profiles at the top wall (8 mm), pane (b) at the vertical center (4 mm), and pane (c) at the bottom (0 mm). Each plot contains three potential profiles obtained in the concentration compartment adjacent to CEM, three in the concentration compartment adjacent to AEM, and three in the desalination channel. The legend in the graphs describes the exact position of the measurement ports.

The results show that the potential profiles monotonously decrease from the cathodic reservoir to the anodic one, i.e., the highest potentials are reached at the concentration side of the anion-exchange membrane and the lowest at the cation-exchange membrane. This observation agrees with the sense of the connected external field, driving anions across the AEM towards the positively charged electrode and the cations across CEM to the negatively charged electrode. At the same time, the differences (the spacing between profiles) in potentials on the concentrate sides and in the desalination channel reflect the conductivities, and thus, the resistivities, of the concentrate and diluate.

The potential profiles in the concentration compartments mostly monotonously grow or shrink. The electrolytes in these compartments are well conducting and constantly mixed. Significant potential drops occur across the membranes. These drops seem lower on AEM than CEM and are given as the sum of the voltage drops on the membrane and the drop across the polarized layers on the diluate side. Unfortunately, the size of the used electrodes does not allow us to probe the solution in the close vicinity of the membranes and reconstruct the profiles within ion-depleted layers. The measured potential profiles in the diluate channel also decrease from the AEM to the CEM with no apparent dependence on the position along the channel. The exception is the inlet region, with the same potential at all heights. This is given by the arrangement of the flow that enters the system through the tubing and expands into the whole channel.

Interestingly, the potential profiles seem closer at the bottom of the channel when compared to the center and the top wall. The observation suggests a smaller resistance at the bottom than in the upper part of the channel. As stated before, we have evidence that natural convection creates a significant concentration gradient in the vertical direction, accumulating ions at the channel bottom. Their higher concentration means higher conductivity, which explains the lower potential drop across the diluate channel. The fluctuating character of the measured data (caused by temporal fluctuations) reflects the possible action of the overlimiting convection and the high sensitivity of the measured value on the position of the second electrode. 

## 4. Discussion

Our system allowed us to experimentally reconstruct electric potential profiles developing in a diluate channel under various experimental conditions. The system was operated in a single-path regime, i.e., no solution recirculation was employed. The major operating parameter was a current load that, under a constant electrolyte flow rate, determined the degree of the solution desalination. The desalination degree (DD) enables us to transfer our results onto other systems. The desalination degree intrinsically dictates the concentration changes in the diluate channel, which in turn, control the local conductivities, and thus, indirectly, the electric potential distribution. The used currents of 10, 20, 30, 40, and 50 mA yielded desalination degrees of 20, 40, 70, 95, and 100%, as derived from Figure 6. Since the size of the measurement electrodes was around 1 mm, unfortunately, we could not reconstruct the electric potentials in the close vicinity of the membranes, where other phenomena also affect the potential distribution.

Our major results can be concisely summarized into the following points: (i) The potential profiles obtained for DD below 70% show minimal variation along the center of the main channel and in the vertical direction. The potential drops across the membranes do not grow along the diluate channel; they only reflect the current load applied to the system, i.e., they are larger at 30 mA than at 10 mA. (ii) DD above 70%, i.e., close to the complete desalination, is characterized by an increased fluctuation of the measured potentials in the desalination channel. The fluctuation scales with the applied current and is most probably caused by overlimiting convection (electroconvection and natural convection) that occurs in the system under these conditions. The potential drops across membranes increase substantially and grow along the channel. This observation suggests that the conditions providing DD close to 100% significantly increase the system resistance. (iii) At high DD, the potential drop on the concentrate side is small, given by the presence of a well-conducting solution. The potential drop across the diluate channel is much larger, despite the ion-depleted layers not being included (the limitation of our method due to the electrode size). Comparing the potential profiles obtained at different vertical layers indicated that the concentration varies in the vertical direction, i.e., the resistance at the channel bottom is smaller than that at the top. 

In future research, we intend to modify our experimental setup so that the ion-depleted zone will be amendable for potential reconstruction. This feature will allow us to better describe the situation in the diluate channel under various operating conditions.

## Data Availability

Not applicable.
